# Hypertriglyceridemic Waist and Metabolic Abnormalities in Brazilian Schoolchildren

**DOI:** 10.1371/journal.pone.0111724

**Published:** 2014-11-14

**Authors:** Flávio Ricardo Guilherme, Carlos Alexandre Molena-Fernandes, Luzia Jaeger Hintze, Maria Teresa Martins Fávero, Roberto Kenji Nakamura Cuman, Wilson Rinaldi

**Affiliations:** 1 Laboratory of Inflammation, Department of Pharmacy and Pharmacology, State University of Maringa, Maringa, Parana, Brazil; 2 Multiprofessional Nucleous of Obesity Studies, Department of Physical Education, State University of Maringa, Parana, Brazil; 3 Department of Physical Education, State University of Maringa, Parana, Brazil; IPO, Inst Port Oncology, Portugal

## Abstract

**Objective:**

To identify the prevalence of hypertriglyceridemic waist (HTW) phenotype and its association with metabolic abnormalities in schoolchildren.

**Methods:**

A cross-sectional study, with a sample of 241 students aged 10 to 14 years from public schools (4 schools) and private (2 schools) from Paranavai town, in Parana State, Brazil. Anthropometric variables (weight, height, waist circumference) and levels of triglycerides, total cholesterol, HDL-C, non-HDL and LDL-C were analyzed. In statistical tests of Pearson partial correlation and multivariate logistic regression, considering p<0,05.

**Results:**

The prevalence of HTW was 20,7% among schoolchildren, 14,1% in males and 6,6% among females with higher proportions aged 10–12 years old. Multivariate analysis indicated that the students who attended private schools were nearly three times more likely (95% CI: 1,2–5,6), to be diagnosed with HTW compared with those who attended public schools (p = 0,006), and LDL-C was the only metabolic variable positively associated with the outcome (p = 0,001), where the students categorized with elevated serum levels had odds 4,2 times (95% CI: 1,6–10,9) having the HTW compared to students in appropriate levels.

**Conclusion:**

This study showed higher prevalence of hypertriglyceridemic waist phenotype in students when compared to prospective studies in Brazil and worldwide. It also showed that the only metabolic alteration associated with HTW phenotype was LDL-C (low density lipoprotein).

## Introduction

Obesity has become a major global health problem in recent decades. Recent data have shown a substantial increase in cases of overweight and obesity in children and adolescents during the past twenty years [1]. The increase in food intake and lack of physical activity, associated with obesity contribute to at least 300,000 deaths per year in the United States. The leading cause of death worldwide among adults are cardiovascular diseases, usually progressive that have their roots in the early years of life [2], [3].

In Brazil these diseases are the leading cause of death in the population in all regions [4]. Childhood and adolescence are important stages in this process, since it is a time of biological changes in the human body, and in addition, adolescents start adopting standards and independent behavior that influence the risk of these diseases [5].

The excess body weight, mainly in the abdominal region, besides being a major risk factor for cardiovascular disease, has been associated with metabolic abnormalities, hypertension, as dyslipidemia, insulin resistance, hyperinsulinemia and diabetes [6], [7].

The hypertriglyceridemic waist phenotype (HTW) is represented by the simultaneous presence of high waist circumference and higher levels of serum triglycerides. That besides being an indicator of visceral obesity and metabolic triad, has been suggested as a predictor of the atherogenic metabolic triad (hyperinsulinemia, elevated apolipoprotein B levels and high concentrations of small and dense low-density lipoprotein (LDL-C), and the metabolic syndrome [8], [9].

The identification of metabolic abnormalities in the young population is essential for the monitoring of those individuals to prevent premature development of cardiovascular diseases and all their related consequences.

Given the above and the few studies published in Brazil on HTW phenotype in children and adolescents population, this study aimed to identify the prevalence of HTW phenotype and its association with metabolic abnormalities in Brazilian schoolchildren.

## Methodology

This cross-sectional survey was conducted in the months of July and August 2013. The sample, consisting of the school population between 6th and 9th degrees aged 10 to 14 years, from public schools (4 schools) and private (2 schools) from Paranavaí town, in Paraná State, Brazil. The classes were chosen by systematic random sampling in three stages: 1) drawing one school from each region of the town; 2) drawing the classes in each school, 3) inviting all schoolchildren from the drawn classes and explanations about the study. Parents received a letter explaining the purpose of the study and were asked for written consent for their child's participation.

The sample calculation resulted from the number of total population analyzed (n = 4,540). Specifically, to investigate the HTW phenotype, the sampling design was defined based on the prevalence of 7.2% of the outcome according to the latest study published in Brazilian adolescents [10]; confidence level equal to 95% and sampling error of 3.5%, estimating the minimum number of 215 students. The assessments were performed only on those students who accepted to participate in the study and presented the Statement of Consent signed by the respondents (n = 248). Seven of these subjects were excluded: 1) different age from 10 to 14 years, 2) non-completion of all assessments. The final sample consisted of 241 children and adolescents, being 136 boys and 105 girls. The margin of sampling error calculated retrospectively, was 3.4% below the value set a priori (3.5%).

The assessments were conducted during school time by trained assessors using calibrated equipment. Height was measured with wall stadiometer (Wisoâ, Brazil) with a resolution of 0.1 cm, and body mass using a digital balance (G-Tech) with a maximum capacity of 150 kg and a resolution of 100 grams. The assessed subject wore only school uniform, without coat or objects in pockets. BMI (kg/m2) was used to classify schoolchildren with appropriate weight and overweight [11]. Data from children with low weight (0.3%, n = 1) were included in the category of appropriate weight.

Waist circumference was measured using an unstretched tape meter (Gullikâ, Brazil), without any pressure to body surface, applied immediately above the iliac crests, and measurements and recorded to the nearest 0.1 cm. For classification of abdominal obesity (central), we used the cutoff point of P ≥75° for all ethnic groups [12].

For biochemical analyzes were collected samples of 10 ml of venous blood in the anti-cubital vein after fasting period of at least 10–12 hours between 8.00 and 9.30am in a clinical analysis laboratory in town. The samples were properly collected and analyzed on the same day of collection. Serum levels of total cholesterol, high density lipoprotein cholesterol (HDL-C), non-high-density lipoprotein cholesterol (non-HDL-C), low density lipoprotein cholesterol (LDL-C), triglycerides and fasting glycemia were determined by enzymatic methods with Gold Analisa kit, according to specifications of the manufacturer. The values of total cholesterol <150 mg/dL, LDL-C <100 mg/dL, HDL-C ≥45 mg/dL, non-HDL-C <123 mg/dl, triglycerides <100 mg/dL, fasting glycemia <100 mg/dl were considered adequate [13]–[15]. The HTW phenotype was defined by the simultaneous presence of abdominal obesity and elevated levels of serum triglycerides (≥100 mg/dL) [16].

In the statistical analysis, the test of Kolmogorov-Smirnov test was used to identify the normality of data distribution. We used the Mann-Whitney's U test for non-parametric independent samples and Student's t test for parametric independent samples to compare anthropometric and biochemical characteristics between the groups with and without the hypertriglyceridemic waist (HTW) phenotype.

In order to analyze the relationship of biochemical and anthropometric variables was performed Pearson partial correlation test with adjustments for age and gender.

Multivariate logistic regression was performed determining the *odds ratio* (OR) and respective confidence intervals (95%), in order to examine the independent association of HTW phenotype (dependent variable) and the independent variables. The criterion for inclusion of independent variables in the multivariate model was an association level of p≤0.20 with the dependent variable, by the chi-square test.

Analyses were performed using the *Statistical Package for Social Science* (SPSS), version 20.0, considering p<0.05. This study was approved by the Ethics Committee in Research of the State University of Maringa, under number 353.552 according to the Declaration of Helsinki.

## Results

Students diagnosed with HTW phenotype showed averages significantly higher of body mass, body mass index, waist circumference, cholesterol, non-HDL-C and triglyceride levels than students without the presence of HTW (p<0.001). The age, height, blood glucose, HDL-C and LDL-C averages were similar between all the groups (Table 1).

**Table 1:** Metabolic Characteristics according to hypertriglyceridemic waist (HTW) phenotype of schoolchildren from Paranavaí town, Paraná state, Brazil, 2013.

	Average ± DP		
Variables	HTW+ (n = 50)	HTW- (n = 191)	p-value
Age (years)	12,3±1,1	12,3±1,2	0,808^b^
Mass (kg)	68,8±9,5	49,2±11,3	<0,001^a^*
Height (cm)	1,62±0,1	1,58±0,1	0,128^b^
BMI (kg/m2)	26,14±2,7	19,67±3,31	<0,001^b^*
WC (cm)	92,6±7,3	71,3±9,4	<0,001^b^*
Glycemia	83,1±13,9	78,7±17,0	0,348^b^
Cholesterol	259,6±31,0	202,9±43,5	<0,001^a^*
HDL-C	48,2±7,4	49,4±6,8	0,634^b^
Non-HDL-C	211,4±29,8	153,4±41,6	<0,001^a^*
LDL-C	83,0±46,7	56,8±37,1	0,063^b^
Triglycerides	127,7±68,2	83,3±38,5	<0,001^b^*

Test t (a), Mann Whitney U Test (b); *p value<0,001. HTW+: presence of phenotype; HTW-: absence of phenotype; BMI: body mass index, WC: waist circumference, HDL-C: high density lipoprotein cholesterol, Non-HDL-C: non-high-density lipoprotein cholesterol; LDL-C: low density lipoprotein cholesterol. HTW phenotype was defined by the simultaneous presence of high waist circumference (≥75th percentile for age and sex) and serum levels of high triglycerides (≥100 mg/dL).

Analyzing the relationship between studied variables, it was found that the two anthropometric indicators were strongly correlated (r = 0.87 p<0.001), indicating collinearity between them. The anthropometric variables showed very weak correlations with all the biochemical variables, and the only significant relationship was between waist circumference and triglycerides (r = 0.16 p<0.001). The biochemical variables showed weak to moderate correlations with each other, with the values of the coefficients ranging from -0.09 to 0.41 (Table 2).

**Table 2:** Pearson Partial Correlation (r) for studied variables, with adjustment for age and gender, of Paranavai schoolchildren, Paraná State, Brazil, 2013.

Variables	WC	BMI	Glycemia	HDL-C	Non-HDL-C	LDL-C	TG
**WC**	-	-	-	-	-	-	-
**BMI**	0,87*						
**Glycemia**	−0,07	0,06	-	-	-	-	-
**HDL-C**	−0,10	−0,12	−0,09	-	-	-	-
**Non-HDL-C**	0,13	0,09	0,33*	0,16*	-	-	
**LDL-C**	0,09	0,06	-0,08	0,12	0,21*	-	-
**TG**	0,16*	0,12	0,17*	0,01	0,41*	0,22*	

BMI: body mass index, WC: waist circumference, HDL-C: high density lipoprotein cholesterol, Non-HDL-C: non-high-density lipoprotein cholesterol; LDL-C: low density lipoprotein cholesterol, TG: triglycerides. * Significant correlations at p<0.001 level.

In exploratory analysis of data, a higher proportion of male schoolchildren (56.4%) was observed. Between the two age ranges adopted in the study (10–12 and 13–14 years), 55.6% of the sample matched the first classification. When checking the presence of CHT phenotype in the sample, 20.7% (n = 50) had a confirmed diagnosis. The isolated prevalence of CHT phenotype components in students was 37.8% (n = 91) for increased waist circumference and 20.7% (n = 50) for high triglycerides serum levels. The data referred to these results, can be found in Data S1.

Then, multivariate analysis by logistic regression showed that the variables associated with the presence of the HTW were gender (p = 0.025) indicating that girls had 0.5 times more likely (CI: 95% 0, 2 to 0.9) of having HTW compared to boys, age (p = 0.009), with students aged 13–14 years had 0.4 times more likely (CI: 95% 0.2–0.8) in comparison to those of 10–12 years, network (p = 0.006) showed that students who attended private schools were nearly three times more likely (95% CI: 1.2 to 5.6), to be diagnosed with HTW compared those who attended public schools and finally LDL-C (p = 0.001) indicated that students classified with elevated serum levels had 4.2 times more likely (95% CI: 1.6 to 10.9) to have the HTW phenotype in relation to students at appropriate levels. BMI, cholesterol and non-HDL-C were not associated with the dependent variable (Table 3).

**Table 3:** Prevalence of hypertriglyceridemic waist (HTW) phenotype and individual components of schoolchildren from Paranavai town, in Parana State, Brazil, 2013.

Variables		Adjusted Odds ratios (IC 95%)
Gender	Boys	1
	Girls	0,5 (0,2–0,9)*
Age	10–12 years	1
	13–14 years	0,4 (0,2–0,8)*
Net	Public	1
	Private	2,7 (1,2–5,6)*
BMI	Eutrophics	1
	Overweight	1,2 (0,6–2,7)
	Obesity	2,1(0,8–5,4)
Cholesterol	Adequate	1
	Inadequate	3,0 (0,3–30,2)
Non HDL-C	Adequate	1
	Inadequate	2,0 (0,6–6,3)
LDL-C	Adequate	1
	Inadequate	4,2 (1,6–10,9)*

BMI: Body Mass Index; Non-HDL-C:non-high-density lipoprotein cholesterol;LDL-C: low density lipoprotein cholesterol. *Significant values p≤0,05.

## Discussion

This study is one of the first in Brazil that observed the HTW phenotype in students of both genders in public and private schools. The prevalence was 20.7% and 14.1% of the total cases were identified in males and 6.6% among females, when the percentage within the genders were analyzed, 25% of boys and 15.2% of girls showed the HTW phenotype.

The first national study of adolescents in Brazil conducted in Minas Gerais [17] found a prevalence of 2.6% of HTW phenotype in female adolescents aged 14 to 19 years, a value far below that the one seen in this study with the girls. In a recent survey of teenagers of both sexes in the state of Bahia-Brazil [10] the prevalence was 7.2% of the total sample, again showing that the values are lower than those shown in school from Paranavaí town, in Paraná State.

Other studies performed elsewhere as in Tehran [16] and Iran [18] with the children and young population showed prevalence of 6.4% and 8.5% respectively of the approaching the prevalence rates found by Brazilian studies published so far, but not with that found in the present study. A likely explanation for the differences found in the prevalence of HTW phenotype would be because of the cutoff points used being different for circumference. In this study an international criterion for all ethnicities [12] which may have been a limitation and bias in the study. Validation of national cut points for this age group are necessary for greater reliability in studies with Brazilian schoolchildren and for a better comparison of results found in studies.

It is important the odds ratio (O.R) of HTW phenotype being higher among male students in the age group of 10-12. In Brazilian study carried recently [10] there was no difference between the sexes, but teenagers aged between 12 and 16 years were twice as likely to have the diagnosis of HTW contrasting the results found in this study.

Another important factor that has been identified, concerns to BMI anthropometric variable, that indicator although it was not significantly associated with the prevalence of HTW, it was the only variable strongly correlated with WC (r = 0.87, p = <0.001). Higher BMI indices may be related directly with excess body fat due to the fact a likely lean mass not be very significant in this population [5], [19], [20], this being an important primary measure and easy to be taken in the school environment to diagnose overweight and obesity among students.

In the case of biochemical variables analyzed in the multivariate model, only LDL cholesterol showed a significant association with HTW phenotype, schoolchildren with inadequate levels had odds 4.2 times (CI: 1.6–10.9) regarding the ones classified as appropriate, confirming the results found for the same variable in other studies [10], [16], [18].

Total cholesterol was not associated with the phenotype CHT probably due to the logistic behavior of data (high prevalence), because 89.6% of the sample were with inappropriate values. This percentage of cases (89.6%) is higher than those found in research with children and adolescents in Brazil and in the world [21]–[23]. It is noteworthy that despite the lack of association, 49 of 50 students diagnosed with CHT phenotype were classified with hypercholesterolemia. Besides being an important metabolic marker, hypercholesterolemia is a major risk factor for cardiovascular disease [24] and the leading cause of death worldwide [3].

Another important variable analyzed was the non-HDL cholesterol, and the results showed no association with the dependent variable, contrasting the only Brazilian study that did similar analysis on this population [10]. Although not associated, it was observed high prevalence (76.3%) of inadequate cases (non-HDL cholesterol ≥123 mg/Dl) in the sample. This high prevalence should be carefully monitored in view of the strong relationship of this measure with the presence of atherogenesis and increased chances of cardiovascular risk [25].

The measures of fasting glycemia and HDL-C (high density lipoprotein cholesterol) were not included in the multivariate model due to weak association seen in exploratory data analysis. The lack of association of HDL-C contradicts the findings of other studies that found a positive association with the HTW [10], [16], [18]. But fasting glycemia behaved in similar way with the research conducted in Bahia and Iran [10], [18]. The prevalence of fasting glycemia was 1.7% (n = 4), and this value, similar to what has been observed in adolescents from different regions of Brazil [7], [26].

Despite the low prevalence found in this study is important and necessary to point out that hyperglycemia is related to obesity, especially visceral, identified from the analysis of the HTW phenotype, it still is positively related to cardiovascular diseases such as, high blood pressure, which in many cases is linked to increased levels of triglycerides in the blood [23]. Thus, its prevention and control is the best way to prevent the emergence of future health complications, especially in metabolism, with the onset of metabolic syndrome.

Given the above, this study showed higher prevalence of hypertriglyceridemic waist phenotype in students when compared to prospective studies in Brazil and worldwide. It also showed that the only metabolic alteration associated with HTW phenotype was LDL-C (low density lipoprotein). Further studies are needed to better clarify what are the metabolic variables related to the phenotype, as there are some discrepancies in the literature. For this, normative values for standardization of criteria and classification of the phenotype in this population should be created so that an accurate comparison between the conducted research is possible.

## Supporting Information

Data S1 Additional data.
(XLS)
