# Correction: Hypertriglyceridemic Waist and Metabolic Abnormalities in Brazilian Schoolchildren

**DOI:** 10.1371/journal.pone.0116445

**Published:** 2014-12-23

**Authors:** The PLOS ONE Staff

The affiliation for the first author is incorrect. Flávio Ricardo Guilherme is not affiliated with #1 but with #3 Department of Physical Education, State University of Maringa, Parana, Brazil.
